# CobB-mediated deacetylation of the chaperone CesA regulates *Escherichia coli* O157:H7 virulence

**DOI:** 10.1080/19490976.2024.2331435

**Published:** 2024-03-19

**Authors:** Linxing Li, Bin Yang, Jing Wang, Yi Wei, Binbin Xiang, Yutao Liu, Pan Wu, Wanwu Li, Yanling Wang, Xinyu Zhao, Jingliang Qin, Miaomiao Liu, Ruiying Liu, Guozhen Ma, Tian Fu, Min Wang, Bin Liu

**Affiliations:** a National Key Laboratory of Intelligent Tracking and Forecasting for Infectious Diseases, TEDA Institute of Biological Sciences and Biotechnology, Nankai University, Tianjin, China; b Key Laboratory of Molecular Microbiology and Technology, Nankai University, Ministry of Education, Tianjin, China; c Nankai International Advanced Research Institute, Nankai University, Shenzhen, China

**Keywords:** CobB, deacetylation, CesA, EspA, virulence, EHEC

## Abstract

Enterohemorrhagic *Escherichia coli* (EHEC) O157:H7 is a common food-borne pathogen that can cause acute diseases. Lysine acetylation is a post-translational modification (PTM) that occurs in various prokaryotes and is regulated by CobB, the only deacetylase found in bacteria. Here, we demonstrated that CobB plays an important role in the virulence of EHEC O157:H7 and that deletion of *cobB* significantly decreased the intestinal colonization ability of bacteria. Using acetylation proteomic studies, we systematically identified several proteins that could be regulated by CobB in EHEC O157:H7. Among these CobB substrates, we found that acetylation at the K44 site of CesA, a chaperone for the type-III secretion system (T3SS) translocator protein EspA, weakens its binding to EspA, thereby reducing the stability of this virulence factor; this PTM ultimately attenuating the virulence of EHEC O157:H7. Furthermore, we showed that deacetylation of the K44 site, which is deacetylated by CobB, promotes the interaction between CesA and EspA, thereby increasing bacterial virulence *in vitro* and in animal experiments. In summary, we showed that acetylation influences the virulence of EHEC O157:H7, and uncovered the mechanism by which CobB contributes to bacterial virulence based on the regulation of CesA deacetylation.

## Introduction

Post-translational modifications (PTMs) of proteins are considered important for survival. Approximately half of all proteins in organisms can be modified by small chemical groups or molecular structures, such as methyl (14 Da), acetyl (42 Da), phosphate (80 Da), oligosaccharide structures (2–3 kDa), or even polypeptide chains (up to 10 kDa). The electron-rich and nucleophilic nature of the lysine side chain makes it suitable for undergoing covalent PTM reactions with diverse substrates.^1^ This residue can potentially be modulated by several PTMs, including acetylation,^2^ propionylation,^3^ malonylation,^4^ crotonylation,^5^ succinylation,^6^ dihydroxybutyrylation,^7^ and the newly discovered lactylation.^8^ The difference between them is that the side-chain molecules that are added are different, and these subtle differences often affect the chemical properties of the modified residues and/or adjacent polypeptide regions. Moreover, acylation can affect the net charge, conformation, and binding properties of proteins, ultimately affecting their function.

Among these modifications, lysine acetylation, which we will refer to as acetylation, is the third most common form of PTM and is well known to regulate protein‒DNA and protein‒protein interactions.^9^ Acetylation can be catalyzed by lysine acetyltransferases (KATs) and lysine deacetylases (KDACs).^10^ In addition to the non-enzymatic mechanism dependent on the concentration of acetyl-coenzyme A, lysine acetylation occurs by the transfer of the acetyl group (-CO-CH3) from acetyl-coenzyme A to amino acid residues by KAT and can be reversed by KDAC. As an example, YfiQ is a Gcn5-like acetyltransferase, and CobB is an NAD^+^-dependent (Sir2-like) lysine deacetylase in *E. coli*.^11–15^ Many studies have indicated that reversible lysine acetylation is involved in the regulation of bacterial virulence. For example, the acetylation of PhoP K201 inhibits the transcription of PhoP-regulated genes to attenuate the magnesium and acid tolerance response, thereby regulating *Salmonella* virulence.^16^ Acetylation of glucosyltransferases GtfB and GtfC, which are enzymatically modified by ActG, can lead to a decrease in their activities, thereby further regulating the virulence and pathogenicity of *Streptococcus*.^17^ Acetylation of the transcription factor RcsB prevents DNA binding, activates flagellar biosynthesis and motility, and increases acid stress susceptibility to regulate the virulence of *E. coli*.^11^

Enterohemorrhagic *Escherichia coli* (EHEC) O157:H7 is an important foodborne pathogen that can cause various diseases, including bloody diarrhea, hemorrhagic colitis, and life-threatening systemic hemolytic uremic syndrome.^18^ As an important EHEC serotype, O157:H7 has a low infectious dose of approximately 50 colony-forming units (CFUs) and is considered the most common cause of pathogenic gastrointestinal diseases in developed countries.^19,20^ This pathogen encodes a T3SS that is required for tight adherence to the intestinal surface and formation of attaching and effacing (A/E) lesions by actin rearrangement of the host cell during infection.^21^ The T3SS of EHEC is encoded by a 35-kilobase locus of enterocyte effacement (LEE) pathogenicity island, which includes many important virulence factors, such as EspA, EspB, and EspD.^22^ In particular, as one of the major EHEC translocator proteins, EspA forms a filamentous conduit along which secreted proteins travel before arriving at the translocation pore in the plasma membrane of the host cell.^23^ EspA has a high tendency to self-oligomerize and is thus maintained in the bacterial cytoplasm by association with a specific chaperone, CesA.^24,25^ In addition, rabbits infected with the *espA* mutant strain showed fewer intestinal A/E lesions than rabbits infected with the wild-type (WT) strain, indicating that the absence of *espA* can significantly reduce bacterial virulence.^26^ The virulence of EHEC is mainly regulated by environmental signals in the gut, such as ammonium concentration, biotin, magnesium ions, and serotonin.^27–30^ However, little research has been conducted on the regulation of EHEC O157:H7 virulence by PTMs. In this study, we used a combination of antibody affinity enrichment and liquid chromatography with tandem mass spectrometry (LC‒MS/MS) to analyze the acetylation profile of EHEC O157:H7 in the presence or absence of CobB and found that the chaperone CesA, one of the substrates of CobB, contributes to EHEC virulence by adjusting its acetylation level.

## Results

### CobB is involved in regulating the virulence of EHEC O157:H7

**Figure 1.**
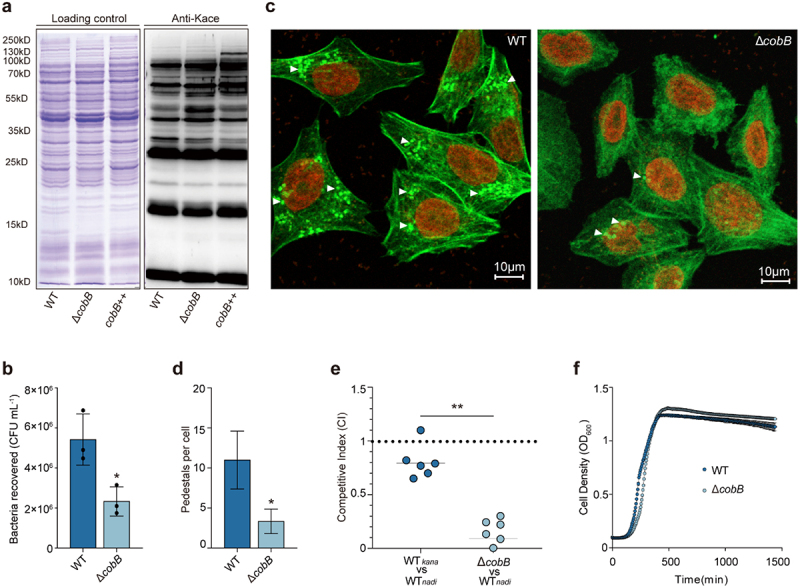
Assays investigation of deacetylation of CobB and its role in virulence regulation.

CobB has been reported to exhibit deacetylation activity in a variety of prokaryotes.^31,32^ To detect the acetylation levels of EHEC O157:H7 *in vivo* and examine whether CobB is a lysine deacetylase in O157:H7, we conducted a Western blot analysis of the wild-type (WT), *cobB* mutant (Δ*cobB*), and *cobB*-overexpressing (*cobB*++) strains using a pan-anti-acetyllysine antibody. The pan-anti-acetyllysine antibody was generated using a synthetic random acetyl-lysine peptide library, which consists of a pool of antibodies that recognize acetylated lysine residues flanked by various sequences, as the antigen, and this analysis revealed the overall abundance of acetylated proteins.^33,34^ At first, we performed SDS‒PAGE and staining with Coomassie Brilliant Blue to ensure equivalent loading of proteins in each lane. The actual blot (Figure 1a) showed that there was a high abundance of acetylated proteins in EHEC O157:H7, and the overall abundance of acetylated intracellular proteins increased when *cobB* was deleted. However, when we overexpressed *cobB* in EHEC O157:H7, the overall abundance of acetylated proteins decreased slightly, especially the acetylation of proteins with a molecular weight near 35 kDa. Our results confirmed that CobB is a deacetylase in EHEC O157:H7. Because the homologous gene *cobB* in *E. coli* strain K12 encodes an NAD^+^-dependent lysine deacetylase,^35^ CobB may also function as a lysine deacetylase in EHEC O157:H7.

(a) Western blot analysis of acetylation in protein lysates from WT, *cobB* mutant (Δ*cobB*) and *cobB*-overexpressing strains (*cobB*++). Coomassie brilliant blue was used as the loading control. (b) Adherence assays of WT and Δ*cobB* to HeLa cells. Data are presented as the mean ± SD (*n* = 3). (c) Detection of A/E lesion formation by WT and Δ*cobB* was evaluated by FAS in HeLa cells 3 hours post infection. The HeLa cell actin cytoskeleton (green) and nuclei of bacterial and HeLa cells (red) are shown. (d) FAS assay quantification of the number of pedestals per infected cell (*n* = 50). Data are presented as the mean ± SD (*n* = 3). (e) Competition assay comparing the colonization ability of Δ*cobB* and WT in the colon of rabbits. The competitive index (CI) is defined as the output ratio of Δ*cobB* to WT divided by the input ratio of WT_*kana*_ to WT_*nadi*_. Each symbol represents the CI of an individual rabbit (*n* = 6). (f) Growth curves of WT and Δ*cobB* in DMEM. Data represent the mean ± SD (*n* = 3). Significant differences were assessed by an unpaired *t* test (b, d) or Mann – Whitney *U* test (e). Error bars represent SD. *p < .05, **p < .01, ***p < .001; n.s. no significant difference.

CobB is an important deacetylase that plays an important role in a wide range of prokaryotes. Thus, we investigated whether CobB affects the virulence of EHEC O157:H7 through an *in vitro* cell adhesion assay. HeLa cells were infected with the EHEC O157:H7 WT and Δ*cobB* strains, and the adherence levels were quantified by evaluating the number of bacteria that adhered to the HeLa cells. The cell adhesion assays results showed that deletion of *cobB* decreased the number of bacteria that adhered to HeLa cells by 2.3-fold compared to that of WT (Figure 1b). Fluorescent actin staining (FAS) assays were performed to evaluate A/E lesions and pedestal numbers – the most important features associated with EHEC adhesion to host cells,^21^ in cells infected with WT or Δ*cobB* strains. The results showed that the pedestal number per cell found for cells infected with Δ*cobB* was significantly reduced (Figure 1c, d) compared with that obtained for WT-infected cells. To verify the contribution of *cobB* to virulence *in vivo*, groups of infant rabbits were orally infected with the WT or Δ*cobB* strains, and intestinal colonization was assessed based on the bacterial load on the colon at 3 days post infection. Competitive infection assays showed that the colonization ability of the Δ*cobB* strain in rabbit colons was reduced by approximately 8-fold compared with that of the WT (Figure 1e). Importantly, the generation of growth curves in DMEM revealed that the WT and Δ*cobB* strains grew at similar rates (Figure 1f), indicating that the decrease in the gut colonization of Δ*cobB* was not due to different growth rate. The above results indicate that the presence of CobB contributes to the virulence of EHEC O157:H7.

### Identification of the substrates of CobB using quantitative acetylome comparison

To better understand the landscape of acetylation in EHEC O157:H7 and how it regulates protein deacetylation to influence bacterial virulence, comparative acetylation proteomics were used to identify the protein modification sites regulated by CobB. First, WT and Δ*cobB* cells were lysed in urea-containing buffer and then digested with trypsin. Immune affinity enrichment of the acetylated peptides was conducted using a pan-anti-acetyllysine antibody conjugated to protein A agarose beads. The enriched peptides were profiled via LC‒MS/MS. The acquired mass spectrometric data were processed and analyzed by PEAKS software to identify acetylated peptides and quantify their relative abundance in the two groups of cells. We considered a peptide to be acetylated if the false recovery rate (FDR) was less than or equal to 1%. Finally, we identified 8978 acetylated peptides and 2128 acetylated proteins (Supplementary Datasets), which accounted for 39% of all proteins in EHEC O157:H7. Among the 2128 acetylated proteins, there were several heavily acetylated proteins, such as RNA polymerase beta prime subunit (40 sites), enolase (ENO) (20 sites), glyceraldehyde-3-phosphate dehydrogenase A (GapA) (16 sites), the HTH-type transcriptional regulator MalT (8 sites), and ribosomal proteins acetylated at multiple sites (Supplementary Datasets).

**Figure 2.**
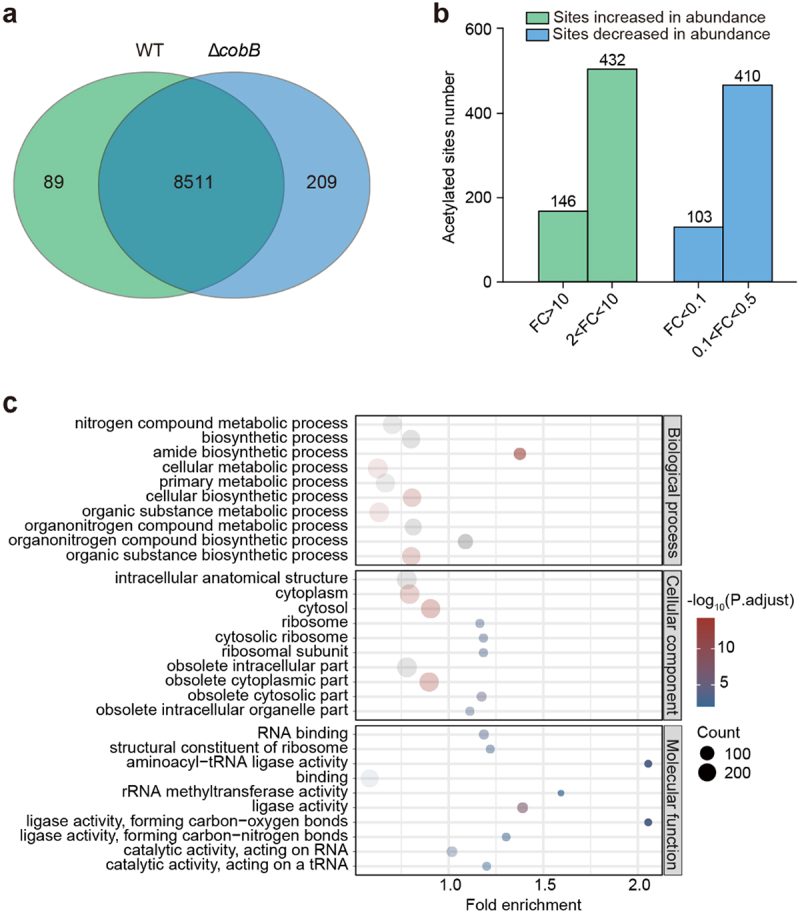
Identification of the substrates of CobB by using quantitative acetylome comparison of Δ*cobB* and WT.

Since CobB has been shown to be a deacetylase enzyme in EHEC O157:H7, we focused on proteins with an increased abundance of acetylation in response to *cobB* deletion. At the peptide level, 8540 acetylated sites overlapped, 120 acetylated sites were only present in WT, and 318 acetylated sites were only present in Δ*cobB* (Figure 2a).

(a) Venn diagram showing the overlap of acetylated sites compared between WT and Δ*cobB*. (b) A column chart showing all the acetylation sites increased or decreased in abundance in the WT and Δ*cobB* strains. The green column represents the sites increased in abundance. The blue column represents the sites decreased in abundance. FC = Fold change. (c) Gene Ontology analysis of acetylated proteins increased in abundance in Δ*cobB*, which are also protein candidates targeted by CobB. These proteins were analyzed for enrichment in three GO ontologies: biological process (upper), cellular component (middle), and molecular function (lower). The *p* value cutoff = 0.05 and *q*-value cutoff = 0.2 were selected as the cutoff criteria. Benjamini and Hochberg correction was used to adjust *p* values.

Among all acetylated peptides present in both WT and Δ*cobB*, we compared those that had a fold change greater than two. As shown in Figure 2b, the abundance of 581 acetylated peptides increased significantly (fold change ≥2.0, *p* < 0.05) in Δ*cobB*; these peptides belonged to 426 proteins. GO analysis showed that these proteins were mainly enriched in metabolism, biosynthesis, and diversity-binding activities mainly occurring in the ribosome and cytosol (Figure 2c). Among these substrates with increased abundance in Δ*cobB*, 11 sites increased more than 100-fold in 11 proteins (Supplementary Datasets), such as K251 of Serine hydroxymethyltransferase (998-fold), K195 of ENO (331-fold) and K138 of GapA (300-fold). In particular, acetylation of the K44 site on the LEE chaperone CesA in the T3SS was increased nearly 705-fold in Δ*cobB*, suggesting that the acetylation of K44 on the CesA protein may be regulated by CobB. Overall, our data revealed that lysine acetylation in EHEC O157:H7 is prevalent and suggests that acetylation-mediated signaling is involved in a broad range of cellular functions.

### Deacetylation of the K44 site of the CesA protein enhanced EHEC O157:H7 adhesion to HeLa cells and bacterial colonization in the colon

A previous study showed that CesA is a chaperone for the translocator protein EspA, and is essential for maintaining the stability of EspA within bacterial cells prior to secretion.^25^ EspA is an important virulence factor that is required to form A/E lesions *in vivo*. Therefore, we speculated that reversible acetylation of the K44 site on CesA may regulate the virulence of EHEC O157:H7 by altering the stability of EspA.

**Figure 3.**
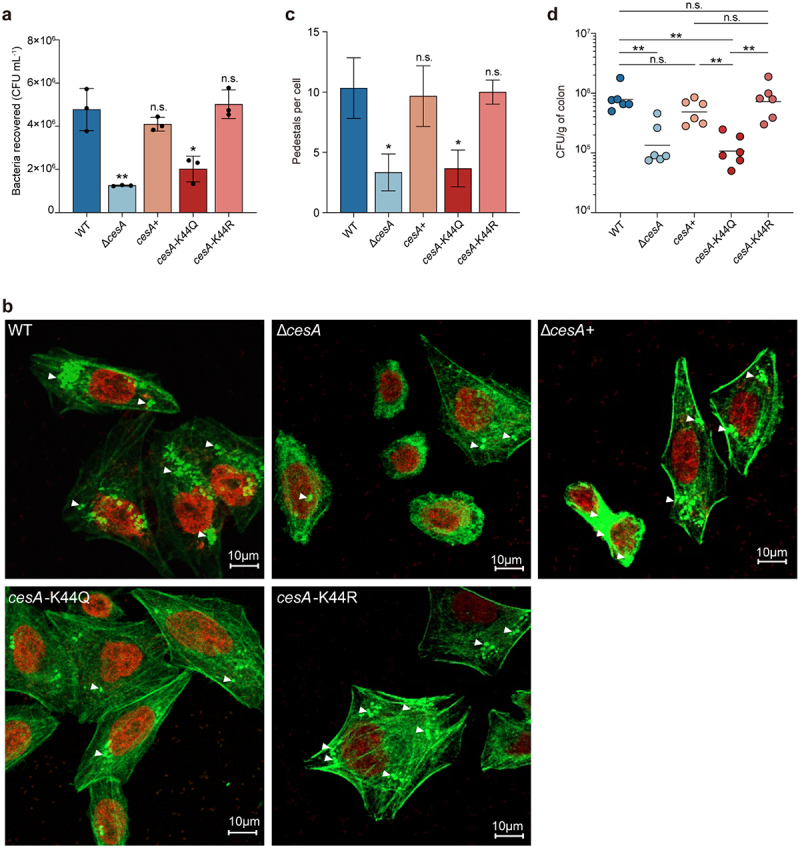
Assays of bacterial adhesion to HeLa cells and bacterial colonization of rabbits.

To test this hypothesis, we performed cell adhesion assays and infant rabbit colonization experiments by generating deletion mutants of *cesA* (Δ*cesA*) to study the role of CesA in regulating the virulence of EHEC O157:H7. As shown in Figure 3a, the ability of Δ*cesA* to adhere to HeLa cells was reduced by 3.8-fold compared to that of the WT strain; however, the level of adherence was restored to that of WT by complementation with *cesA* (Δ*cesA*+). FAS assays further showed that both the percentage of HeLa cells that formed pedestals and the number of pedestals formed per infected cell were significantly reduced by *cesA* deletion (Figure 3b, c). Infant rabbit colonization experiments showed that colonization by the Δ*cesA* strain decreased by approximately 7.8-fold compared with colonization by the WT strain, whereas *in vivo* colonization by the Δ*cesA*+ strain was restored to the level of the WT strain.

(a) Adhesion assays of the WT, Δ*cesA*, *cesA*+, *cesA*-K44Q, and *cesA*-K44R strains to HeLa cells. Data represent the mean ± SD (*n* = 3). (b) Detection of A/E lesion formation by WT, Δ*cesA*, *cesA*+, *cesA*-K44Q, and *cesA*-K44R by FAS of HeLa cells at 3 hours post infection. The HeLa cell actin cytoskeleton (green) and nuclei of bacterial and HeLa cells (red) are shown. (c) FAS assay quantification of the number of pedestals per infected cell (*n* = 50). (d) Colonization assays of the WT, Δ*cesA*, *cesA*+, *cesA*-K44Q, and *cesA*-K44R strains in the colon of rabbits (*n* = 6). Significant differences were assessed by an unpaired *t* test (*a*, *c*) or Mann – Whitney *U* test (*d*). Error bars represent SD. *p < .05, **p < .01, ***p < .001; n.s. no significant difference.

To further study the role of the acetylated K44 site in CesA, we constructed two plasmids (pBlue-*cesA*-K44Q and pBlue-*cesA*-K44R) harboring *cesA* with K44 mutated to Q or R. The K-to-Q (replacement of lysine by glutamine) substitution mimics constitutive acetylation by changing the positive charge to a neutral charge, whereas the K-to-R (replacement of lysine by arginine) substitution mimics the deacetylated state. We then introduced the pBlue-*cesA*-K44Q and pBlue-*cesA*-K44R plasmids into the Δ*cesA* strain, generating the strains *cesA*-K44Q and *cesA*-K44R, respectively. As shown in Figure 3a, d, cell adhesion and infant rabbit colonization assays were performed to examine the role of acetylated K44 in the CesA-mediated regulation of EHEC O157:H7 virulence. The adherence of strain *cesA*-K44Q to HeLa cells was reduced 2.37-fold compared to that of the WT (Figure 3a), and there were fewer A/E lesions in cells infected with *cesA*-K44Q than in cells infected with *cesA*-K44R or the complemented strain Δ*cesA* (+) (Figure 3b, c). Furthermore, the rabbit colonization results showed that the intestinal colonization ability of strain *cesA*-K44Q was significantly decreased compared to that of WT and Δ*cesA* (+), but the intestinal colonization ability of *cesA*-K44R was similar to that of WT and Δ*cesA* (+) (Figure 3d). Overall, the above results indicate that deacetylation of the K44 site on the CesA protein increases the virulence of EHEC O157:H7.

### Deacetylation of the K44 site on CesA improves the stability of EspA

**Figure 4.**
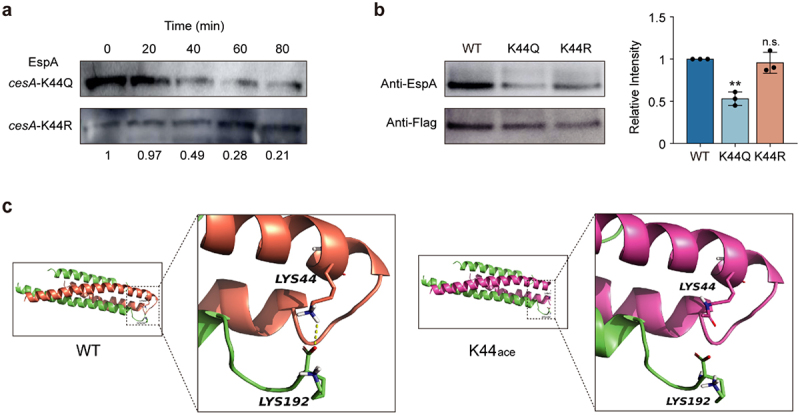
Assays of EspA stability and interaction with CesA.

CesA is critical for maintaining the stability of EspA.^25^ To investigate whether the stability of EspA was influenced by acetylation of the K44 site of CesA, we performed a Western blot analysis of EspA in strains *cesA*-K44Q and *cesA*-K44R for *in vivo* degradation assays. After the cells had grown in DMEM to the exponential phase, protein translation was blocked with 200 mg/mL chloramphenicol, a sample was collected every 20 min, and the EspA protein level was detected using an anti-EspA antibody. As shown in Figure 4a, the amount of EspA protein in the *cesA*-K44Q strain gradually decreased with time, whereas that in the *cesA*-K44R strain remained stable.

(a) Western blot analysis of EspA stability in the *cesA*-K44Q and *cesA*-K44R backgrounds. (b) Western blot analysis of the EspA interaction with CesA-FLAG (WT), CesA-K44Q-FLAG (K44Q), and CesA-K44R-FLAG (K44R) purified by immunoprecipitation. The data are presented as the means ± SDs (*n* = 3). (c) Structural representation of the interactions between K44 of CesA and EspA. The left part is based on the X-ray crystal structure of the CesA-EspA complex (PDB: 1XOU). The right structure was generated through computational modeling. Significant differences were assessed by an unpaired *t* test. Error bars represent SD. *p < .05, **p < .01, ***p < .001; n.s. no significant difference.

Previous studies have shown that CesA binds directly to EspA.^36^ To further verify the acetylation of K44 directly affects the binding affinity between CesA and EspA and ultimately reduces the stability of EspA. We constructed three plasmids expressing CesA-FLAG, CesA-K44Q-FLAG, and CesA-K44R-FLAG and introduced these plasmids into Δ*cesA*, generating the strains *cesA*-Flag, *cesA*-K44Q-Flag, and *cesA*-K44R-Flag, respectively. We then immunoprecipitated CesA-FLAG, CesA-K44Q-FLAG, and CesA-K44R-FLAG from Δ*cesA* using an anti-Flag antibody for Western blot analysis. The amount of EspA that interacted with CesA was detected with an antibody against EspA. The results indicated that the K44Q mutation in CesA caused a marked decrease in EspA binding to CesA, whereas K44R had no such effect (Figure 4b).

The crystal structure of the CesA-EspA complex has been previously determined.^36^ To further examine the possible effects of acetylation on the complex, we performed molecular dynamics simulations of the structures of wild-type EspA and CesA with K44 acetylation. The docking results demonstrated a stable interaction between wild-type EspA and CesA through hydrogen bonds. However, when acetylation was present at the K44 site, the hydrogen bond between the two disappeared (Figure 4c). These results showed that deacetylation of the K44 site in CesA promoted the stability of EspA, which contributed to the virulence of EHEC O157:H7.

### CobB directly interacts with CesA

**Figure 5.**
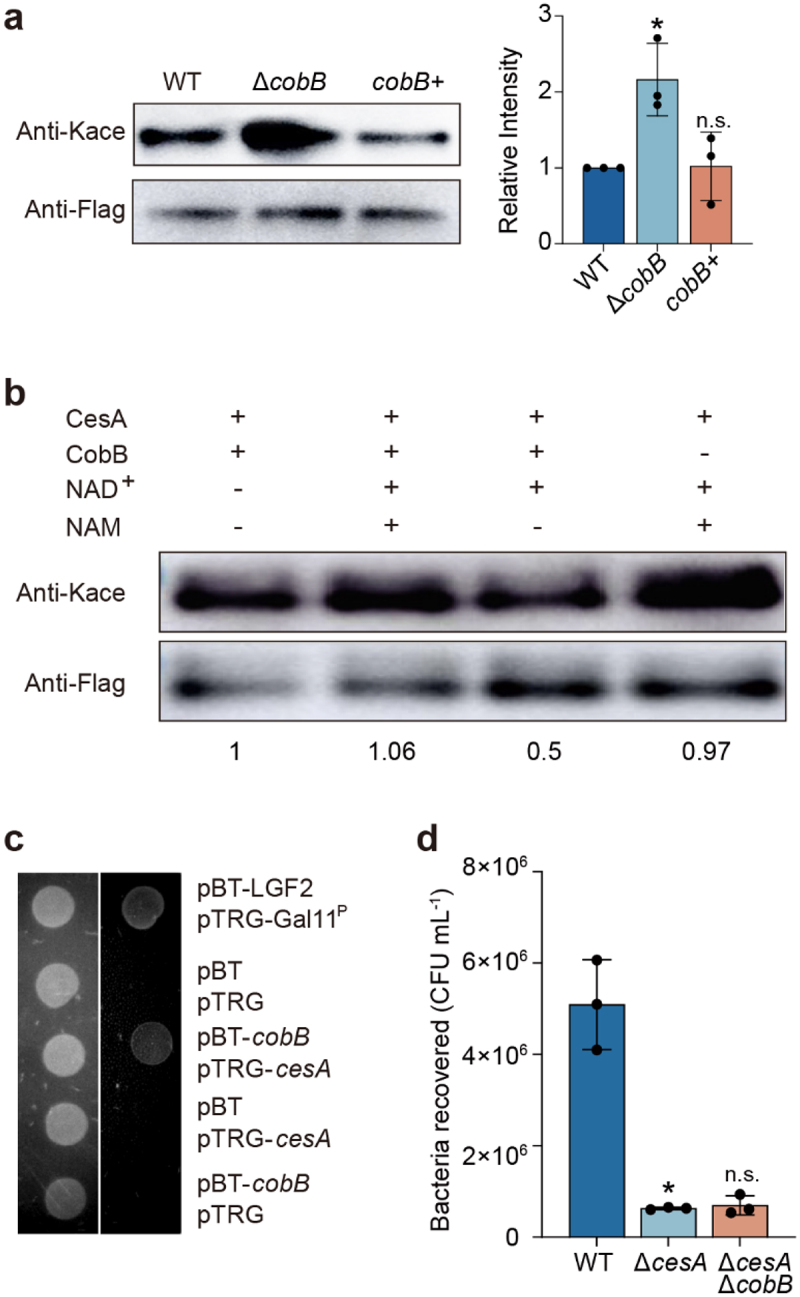
Assays of CesA interactions with CobB.

CobB has been identified as a NAD^+^-dependent lysine deacetylase.^31^ To investigate whether deacetylation of the CesA protein was directly mediated by CobB in EHEC O157:H7, we immunoprecipitated CesA-FLAG from Δ*cesA*, double-mutant Δ*cobB*Δ*cesA* and *cobB* complemented strain Δ*cobB* (*+*) Δ*cesA*, and then analyzed the acetylation level of CesA-FLAG by Western blot using a pan-anti-acetyllysine antibody. The results showed that the acetylation level of the CesA-FLAG protein in Δ*cobB*Δ*cesA* was greater than that in Δ*cesA* (Figure 5a). To further test whether CobB could regulate the acetylation level of CesA *in vitro*, we purified the proteins CesA and CobB and incubated CesA with CobB in the presence or absence of NAD^+^ or NAM *in vitro*. Western blot analysis showed that the acetylation of CesA decreased significantly in the presence NAD^+^, but no significant change in the acetylation of CesA was detected in the presence of NAD^+^ and NAM (Figure 5b).

(a) Western blot and quantitative analysis of the acetylation level of the CesA-FLAG protein immunoprecipitated from strains *cesA*-Flag (WT), Δ*cobBcesA*-Flag (Δ*cobB*) and Δ*cobB* (*+*) *cesA*-Flag (Δ*cobB+*). “Anti-Kace” means “pan-anti-acetyllysine antibody”. (b) Western blot of CesA, incubated with CobB in the presence of NAD^+^ or NAM as inhibitor. Ten hours of incubation at 25°C revealed that NAD^+^-dependent CobB can catalyze deacetylation with CesA. (c) Bacterial two-hybrid assays for the interaction between CobB and CesA. Left panel: plate minus streptomycin (str) and 3-AT. Right panel: plate plus 12.5 mg/mL str and 15 mM 3-AT. Co-transformants containing pBT-LGF2 and pTRG-Gal11^P^ were used as positive controls. A co-transformant containing pBT and pTRG was used as a negative control. Each unit representing the corresponding co-transformant in the plates is indicated in the figure. (d) Adhesion assays of the WT, Δ*cesA*, and Δ*cesA*Δ*cobB* strains to HeLa cells. Data are presented as the mean ± SD (*n* = 3). Significant differences were assessed by an unpaired *t* test. Error bars represent SD. *p < .05, **p < .01, ***p < .001; n.s. no significant difference.

Furthermore, to confirm the interaction between CesA and CobB, a bacterial two-hybrid assay was conducted. Two plasmids were constructed: pBT-*cobB*, which expresses the bait protein (λcI-CobB fusion protein), and pTRG-*cesA*, which expresses the target protein (RNAPα-CesA fusion protein). If bait and target proteins that are cotransformed into the reporter strain interact, RNA polymerase is recruited to the promoter of the *HIS3* reporter gene, where it activates transcription, which allows the growth of the reporter strain in the presence of 3-amino-1,2,4-triazole (3-AT).^37–39^ As shown in Figure 5c, cell growth was observed in the presence of 5 mM 3-AT when both pBT-*cobB* and pTRG-*cesA* were cotransformed into the *E. coli* selection host. No cell growth was detected in the negative controls. The results confirmed that CobB interacts with CesA.

Additionally, we investigated whether CobB influences bacterial virulence via CesA. We used WT, Δ*cesA* and Δ*cobB*Δ*cesA* to perform cell adhesion assays with HeLa cells. As expected, the adherence of Δ*cesA* was significantly lower than that of the WT. However, there was no further decrease in cell adherence in Δ*cobB*Δ*cesA* compared to that in Δ*cesA* (Figure 5d). These data indicate that CobB regulates the virulence of EHEC O157:H7 by deacetylating CesA.

## Discussion

CobB, a sirtuin-like deacetylase, plays an important role in the physiological functions of many bacteria. For example, CobB deacetylates acetyl-CoA synthase (Acs) to alter cellular energy metabolism processes.^14^ Additionally, CobB regulates chemotaxis by deacetylating the chemotaxis response regulator protein, CheY.^40^ In addition, CobB affects the OAT activity of NhoA, an N-hydroxyarylamine O-acetyltransferase, and can help maintain the catalytic activity of TopA, a topoisomerase I, to reduce DNA supercoiling.^41^ Furthermore, CobB-mediated deacetylation attenuates the virulence of *Yersinia pestis* and reduces the virulence of *V. cholerae* in *Drosophila*.^32,42–44^ However, whether CobB is involved in regulating the virulence of EHEC O157:H7 has not yet been reported. In the present study, we systematically identified many proteins that could be regulated by CobB in EHEC O157:H7 using acetylation proteomics and discovered that CobB promotes the virulence of O157:H7 by reversing the acetylation at the K44 site on CesA, a chaperone for EspA. Given that CobB is a widely conserved protein in bacteria^31^ and that CesA and EspA are present in many other EPEC and EHEC serotypes, as revealed by genome sequence analysis, acetylation-mediated control by CobB may also contribute to the virulence of other EHEC and EPEC strains.

In addition to CesA, many of the central metabolic pathway enzymes identified here were lysine acetylated, suggesting that acetylation targets a broad range of fundamental cellular processes, ranging from the control of binding to metabolic pathways. Proteins such as enolase (ENO), glyceraldehyde-3-phosphate dehydrogenase A (GapA), and 2-isopropylmalate synthase (IPMSs) are key enzymes in the glycolytic or biosynthetic pathway^45–47^ and are hyperacetylated in Δ*cobB*. ENO is a glycolytic enzyme that catalyzes the dehydration of 2-phospho-d-glycerate to form phosphoenolpyruvate and the reverse of gluconeogenesis. Previous studies have shown that acetylation of ENO at K326 is regulated by CobB, thereby influencing cell growth.^48^ The acetylation of K195 on ENO was significantly higher in Δ*cobB*, suggesting a markedly stronger regulation of cell growth by the acetylation of K195. MalT is an HTH-type transcriptional activator that controls the expression of the maltose regulon in *E. coli*,^49^ and its activity is strictly dependent on the presence of ATP.^50^ We observed that acetylation at K25 occurs in the ATPase domain of MalT, which is important for ATP binding.^49^ This finding indicates that acetylation likely modulates the ability of MalT to bind ATP, which may influence maltose uptake and utilization.^49^ Moreover, ribosomal proteins acetylated at multiple sites probably affect the ribosome assembly and activity.^51^ Collectively, these results suggest that lysine acetylation of proteins may play an important role in a variety of bacterial physiological processes. Further studies are warranted to fully understand the role of lysine acetylation in the regulation of EHEC virulence.

## Materials and methods

### Ethics statement

All animal experiments were performed in accordance with the standards set forth in the Guide for the Care and Use of Laboratory Animals. The experimental protocols were approved by the Institutional Animal Care Committee of Nankai University.

### Strains, plasmids, primers, and media

All wild-type strains and their derivatives, as well as the plasmids, and primers used in this study, are described in Supplementary Table S1 and Supplementary Table S2. Mutant strains were generated using the λ-red recombinase system. Briefly, the pKD46 plasmid was introduced into the WT strain to express three proteins (Exo, Beta, and Gam) required for homologous recombination. Kanamycin- and chloramphenicol-resistant fragments were amplified using the pKD3 or pKD4 plasmid as a template. All the strains were cultured overnight in LB or DMEM at 37°C.

### Bacterial adhesion to HeLa cells

Adherence assays were performed as described previously.^30^ Briefly, bacteria were added to HeLa cells at a multiplicity of infection (MOI) of 10. At 3 h post-infection, the HeLa cells were washed three times with PBS to remove non-adherent bacteria and were treated with 1 mL of 0.1% Triton X-100 for 5 min. The gradient-diluted cell lysates were spread on agar plates and the bacterial colonies were counted. The adhesion efficiency of different strains was compared by calculating the number of bacteria per mL.

### Growth curve

The overnight strains cultured in LB broth at 37°C and 180 rpm were transferred to 50 mL of LB broth. Subsequently, a 200 μl aliquot was added to a 96-well plate. The absorbance at OD_600 nm_ was measured and recorded every 30 min for 24 h at 37°C and 180 rpm automatically using a multifunctional microplate tester (TECAN Spark, Shanghai, China). Three independent experiments were performed, and the results were analyzed.

### Intestinal colonization assay

Three-day-old female New Zealand white rabbits were housed with their mother. Infant rabbits were infected as described previously.^52^ Overnight growing bacteria were centrifuged at 5500 × g and resuspended in PBS at a 10-fold concentration. Each rabbit was gavaged with 100 µL of bacterial solution containing 10^9^ CFUs of bacteria at the logarithmic stage of growth. After 3 days of infection, length of 1–3 cm of colon tissue was separated and weighed, after which the homogenates were diluted and plated on LB agar. The attachment efficiency *in vivo* was determined by counting the number of CFUs per gram of the colon.

For the *in vivo* competition assay, WT_*nadi*_, WT_*kana*_, Δ*cobB* and WT_*nadi*_ strains were grown overnight at 37°C in LB broth. Approximately 10^5^ CFUs of the WT_*nadi*_ strain were mixed with the WT_*kana*_ at a ratio of 1:1. Similarly, 10^5^ CFUs of the Δ*cobB* strain were mixed with the WT_*nadi*_ at a ratio of 1:1, and the mixtures were gavaged into six rabbits. The competitive index (CI) was determined as the output ratio divided by the input ratio.

### Mass spectrometry-based label-free quantitative acetylated proteomics

Mass spectrometry analysis was performed blindly using the Applied Protein Technology (Shanghai, China). Briefly, urea (8 M urea, 100 mM Tris/HCl, pH 8.5) buffer was used for sample lysis and protein extraction. Trypsin was then added to the samples for overnight digestion. Acetylated peptides were enriched using a PTMScan Acetyl-Lysine Motif Kit (Cell Signalling Technology, #13416). LC-MS/MS analysis was performed on a timsTOF Pro mass spectrometer (Bruker) coupled to a nanoelute (Bruker Daltonics) for 60 min. The raw MS data for each sample were combined and searched using PEAKS software with the NCBI_EDL933_17828_20210510.fasta protein database for identification and quantification.

### Immunoprecipitation and Western blot

The cells were pelleted by centrifugation at 4°C, washed three times, resuspended in PBS, and subsequently disrupted by sonication. The cell lysates were mixed by rotation with pretreated anti-Flag magnetic beads (MCE, # HY-K0207), and proteins with FLAG tags were obtained according to the manufacturer’s protocol. Purified CesA-Flag protein was analyzed by Western blot using an anti-Flag antibody (Meck, #F1804) and pan-anti-acetyllysine antibody (PTM Bio, #PTM-101).

For Western blot, purified proteins were loaded onto a 4%-12% SDS-polyacrylamide gel electrophoresis (SDS-PAGE) gel and transferred to a polyvinylidene fluoride (PVDF) membrane (Millipore, #IPVH00010). The membranes were blocked with 5% (w/v) skimmed milk powder dissolved in Tris-buffered saline (TBS) containing 0.05% (v/v) Tween 20, incubated for 1 h at room temperature and then incubated overnight at 4°C with anti-Flag and pan-anti-acetyllysine antibody as the primary antibodies at a dilution of 1:10000. Next, goat anti-mouse (Thermo Scientific, #31430) or goat anti-rabbit (Thermo Scientific, #31460) IgG conjugated with horseradish peroxidase (HRP) was added to the blot at a dilution of 1:10000 and incubated. Finally, the signal was detected using an ECL system (Thermo Scientific, USA) and images were acquired using an Amersham Imager 600 system (General Electric).

### Bioinformatic analysis

The GO annotations of the proteins were divided into three broad categories: biological process, cellular component, and molecular function. Enrichment analysis was performed using Fisher’s exact test with all quantified proteins as the background dataset. The Benjamini–‒Hochberg correction for multiple comparisons was further applied to adjust the *p* values. Only functional categories and pathways with *p* values under the threshold of 0.05 were considered significant.

### Molecular docking

Crystal structures were obtained from PDB (PDB:1XOU). The Protein Preparation Wizard module in Maestro v.12.8 was employed for processing the protein structure. The overall structural charges and protonation states were prepared as necessary. The orientation of the water molecules and other functional groups, such as amides and hydroxyls, was checked. The acetylated structure was obtained using AutoDock Vina software for covalent docking. The results were analyzed and visualized using PyMOL software.

### *Degradation of EspA* in vivo

The stability of EspA was determined using *in vivo* degradation experiments. *cesA*-K44Q or *cesA*-K44Q strains grown overnight were inoculated at 1:100 in DMEM at 37°C. When the OD_600 nm_ reached 0.7, translation was blocked with 200 mg/mL chloramphenicol, and 2 mL of the bacterial solution was collected every 20 min and centrifuged at 4°C. The cell pellets were resuspended in radioimmunoprecipitation assay (RIPA) lysis buffer (Solarbio, #R0020) for 10 min. The supernatant was then separated for Western blot analysis.

### In vitro *deacetylation assay*

The purified CobB and CesA-Flag proteins were incubated in reaction buffer [50 mM Tris-HCl, 137 mM NaCl, 2.7 mM KCl, 1 mM MgCl_2_, and 1 mM DTT (pH 8)] with or without 0.5 mM NAD^+^ and in the presence or absence of 10 mM NAM. The mixture was incubated at 25°C for 10 h. The reaction products were separated by SDS‒PAGE and analyzed by Western blot using a pan-anti-acetyllysine antibody.

### Fluorescent actin staining

Fluorescent actin staining was performed as previously described.^53^ Briefly, overnight bacteria grown at 37°C and 180 rpm were subcultured at 1:100 in DMEM. Cultures in the exponential phase were added to HeLa cells grown overnight on sterile coverslips in 6-well plates at a ratio of 1:100 and incubated for 3 h. The coverslips were then washed with PBS and fixed with 4% formaldehyde. After permeabilization with 0.2% Triton-X, the actin filaments of the cells were stained and visualized with fluorescein isothiocyanate-labeled phalloidin, and the cell nuclei of both were observed with propidium iodide. A minimum of 50 HeLa cells were counted to determine the number of A/E lesions formed by each bacterial strain.

### Bacterial two-hybrid assay

The protein–protein interaction between CobB and CesA was detected using the BacterioMatch II Two-Hybrid System Vector (Agilent Technologies; #240065) according to a previously reported procedure.^16^ The *cobB* and *cesA* genes were cloned and inserted into the pBT and pTRG plasmids, respectively. The reporter strain was cotransformed with the recombinant vectors pBT-*cesA* and pTRG-*cobB* and subsequently spotted onto screening media supplemented with 5 mM 3-amino-1,2,4-triazole (3-AT), 12.5 mg/ml tetracycline, 12.5 mg/ml streptomycin, and 25 mg/ml chloramphenicol. A cotransformant containing pBT-LGF2 and pTRG-Gal11^P^ was used as a positive control for growth on the screening medium. A cotransformant containing the empty vector pBT and pTRG was used as a negative control

## Supplementary Material

0311_Revised_Supplementary Information.docx

0311_Revised_Supplementary Datasets.xlsx

## Data Availability

The relevant data are provided in the manuscript (Supplementary Information). MS proteomics data were deposited in the ProteomeXchange Consortium (http://proteomecentral.proteomexchange.org) via the iProX partner repository with the dataset identifier PXD045950.
