# Supplementary material for: CobB-mediated deacetylation of the chaperone CesA regulates Escherichia coli O157:H7 virulence
Source: Gut Microbes. 2024 Mar 19;16(1):2331435. doi: 10.1080/19490976.2024.2331435 (PMC10956630; doi:10.1080/19490976.2024.2331435)
Supplement: 0311_Revised_Supplementary Information.docx [file KGMI_A_2331435_SM8795.docx]

**Supplementary Information for**

**CobB-mediated deacetylation of the chaperone CesA regulates *Escherichia coli* O157:H7 virulence**

**This file includes:**

Supplementary Table 1 to 2

**Other supplementary materials for this manuscript include the following:**

Supplementary Datasets: Mass spectromy results

**Supplementary Table 1**. **Strains and plasmids used in this study.**

| **Strains** | **Genotype or description** | **Source** |
| --- | --- | --- |
| EHEC O157: H7 | Wild-type EHEC serotype O157: H7 strain EDL933 | ATCC* |
| Δ*cobB* | *cobB* deletion mutant in O157 WT; Km^R^ | This work |
| *cobB++* | pTRC99a-*cobB* in O157 WT strain; Ap^R^ | This work |
| BL21(DE3)-CobB | pET28a-*cobB* in BL21(DE3) strain; Km^R^ | This work |
| Δ*cesA* | *cesA* deletion mutant in O157 WT; Cm^R^ | This work |
| Δ*cesA*+ | pBlue-*cesA* in Δ*cesA* strain; Cm^R^ Ap^R^ | This work |
| *cesA*-K44Q | pBlue-*cesA*-K44Q in Δ*cesA* strain; Cm^R^ Ap^R^ | This work |
| *cesA*-K44R | pBlue-*cesA*-K44R in Δ*cesA* strain; Cm^R^ Ap^R^ | This work |
| *cesA*-K44Q-Flag | pBlue-*cesA*-K44Q-Flag in Δ*cesA* strain; Cm^R^ Ap^R^ | This work |
| *cesA*-K44R-Flag | pBlue-*cesA*-K44R-Flag in Δ*cesA* strain; Cm^R^ Ap^R^ | This work |
| *cesA*-Flag | pBlue-*cesA-*Flag in Δ*cesA* strain; Cm^R^ Ap^R^ | This work |
| Δ*cobBcesA*-Flag | pBlue-*cesA-*Flag in Δ*cesA*Δ*cobB* strain; Km^R^ Cm^R^ Ap^R^ | This work |
| Δ*cobB*(*+*)Δ*cesA*-Flag | pBlue-*cesA-*Flag and pACYC184-*cobB* in Δ*cesA*Δ*cobB* strain; Km^R^ Cm^R^ Tet^R^ | This work |
| Δ*cobB*Δ*cesA* | *cobB* and *cesA* double mutant in O157 WT; Km^R^ Cm^R^ | This work |
| Δ*cobB*(*+*)Δ*cesA* | pACYC184-*cobB* in Δ*cesA*Δ*cobB* strain; Km^R^ Cm^R^ Tet^R^ | This work |
| **Plasmids** | **Genotype or description** | **Source** |
| pKD3 | For λ Red recombination; Cm^R^ | Lab collection |
| pKD4 | For λ Red recombination; Km^R^ | Lab collection |
| pTRC99a | A expression vector; Ap^R^ | Lab collection |
| pET28a(+) | T7 Expression vector; Km^R^ | Lab collection |
| pBluescript II SK(+) | A cloning vector; Ap^R^ | Lab collection |
| pTRC99a-*cobB* | pTRC99a plasmid carrying *cobB* gene; Ap^R^ | This work |
| pET28a-*cobB* | pET28a(+) plasmid carrying the *cobB* gene; Km^R^ | This work |
| pBlue-*cesA* | pBluescript II SK(+) plasmid carrying *cesA* gene; Ap^R^ | This work |
| pBlue-*cesA-*Flag | pBluescript II SK(+) plasmid carrying *cesA* gene with Flag; Ap^R^ | This work |
| pACYC184-*cobB* | pACYC184 plasmid carrying *cobB* gene; Tet^R^ | This work |
| pBlue-*cesA*-K44Q | pBluescript II SK(+) plasmid carrying *cesA* gene(lysine of site 44 were replaced by glutamine); Ap^R^ | This work |
| pBlue-*cesA*-K44Q-Flag | pBluescript II SK(+) plasmid carrying *cesA* gene(lysine of site 44 were replaced by glutamine) with Flag; Ap^R^ | This work |
| pBlue-*cesA*-K44R | pBluescript II SK(+) plasmid carrying *cesA* gene(lysine of site 44 were replaced by arginine); Ap^R^ | This work |
| pBlue-*cesA*-K44R-Flag | pBluescript II SK(+) plasmid carrying *cesA* gene(lysine of site 44 were replaced by arginine) with Flag; Ap^R^ | This work |
| pBT | pBT bait plasmid for Bacterial two-hybrid system | Stratagene |
| pTRG | pTRG prey plasmid for Bacterial two-hybrid system | Stratagene |
| pBT-LGF2 | Bacterial two-hybrid system control plasmid | Stratagene |
| pTRG-Gal11^P^ | Bacterial two-hybrid system control plasmid | Stratagene |
| pBT-*cobB* | pBT plasmid carrying the *cobB* gene; Cm^R^ | This work |
| pTRG-*cesA* | pTRG plasmid carrying the *cesA* gene; Tet^R^ | This work |

**Supplementary Table 2. Primers used in this study (5'-3').**

| Δ*cobB* | F | GCGGGAGGAATGCGTGGTGCGGCCTTCCTACATCTAACCGATTAAACAACAGAGGTTGCT**CATATGAATATCCTCCTTAG（Cm）** |
| --- | --- | --- |
| Δ*cobB* | R | CGGTATTGTGGTCCGCTTGTAGGCCTGATAAGCGTAGCGCATCAGGCAATGCTTCCTGCT**GTGTAGGCTGGAGCTGCTTC（Cm）** |
| Δ*cobB*-Identify | F | TACCTGTAGCTCGTGTTCCGCG |
| Δ*cobB*-Identify | R | CGCTTGTAGGCCTGATAAGCGTAG |
| Δ*cesA* | F | TGTGATGCGGCAATTAGGACTATAGAGATAATGCGAATCAGGATTAATAATAAATAGAGG**GTGTAGGCTGGAGCTGCTTC（Ka）** |
| Δ*cesA* | R | CCATCATGATTGGATAATTAATTAATTTATAATAGTTATAGTCTATTTTATTAAAAATTG**CATATGAATATCCTCCTTAG（Ka）** |
| Δ*cesA*-Identify | F | ATTGCGTGTGATGCGGCAATT |
| Δ*cesA*-Identify | R | ACTGACCTGATTGACACCGGT |
| pBlue-*cesA-Hind* III | F | CCCAAGCTTATGAGTATTGTGAGCCAAACAAGA |
| pBlue-*cesA*-*BamH* I | R | CGCGGATCCTCATACTATTTTTCTATTATTTCTATTCCGttgattcattg |
| pBlue-*cesA*-Identify | F | TAATACGACTCACTATAGGG |
| pBlue-*cesA*-Identify | R | ATTAACCCTCACTAAAGGGA |
| pBT-*cobB*-*Not* I | F | ATAAGAATGCGGCCGCatgctgtcgcgtcggggtcat |
| pBT-*cobB*-*Xho* I | R | CCGCTCGAGttataatcccttcagcaacttttcaacaaattctg |
| pTRG-*cesA*-*BamH* I | F | CGCGGATCCatgagtattgtgagccaaacaaga |
| pTRG-*cesA*-*Xho* I | R | CCGCTCGAGtcatactatttttctattatttctattccgttgattcattg |
| pBT-*cobB*-Identify | F | GAGCCAGGTGATTTCTGCATAGCC |
| pBT-*cobB*-Identify | R | CAGATGTAGGTGTTCCACAGGGTAG |
| pTRG-*cesA*-Identify | F | TGGCTGAACAACTGGAAGCT |
| pTRG-*cesA*-Identify | R | ATTCGTCGCCCGCCATAA |
| Some plasmids were synthesized by GENWIZ | | |
